# Molecular cloning, expression and adhesion analysis of silent *slpB* of *Lactobacillus acidophilus* NCFM

**DOI:** 10.1186/s13568-018-0631-2

**Published:** 2018-06-23

**Authors:** Yuxing Guo, Xiangyue Li, Yao Yang, Zhen Wu, Xiaoqun Zeng, Fawze Nadari, Daodong Pan

**Affiliations:** 1 Department of Food Science & Nutrition, Ginling College, Nanjing Normal University, Nanjing, 210097 People’s Republic of China; 2 Key Laboratory of Animal Protein Deep Processing Technology of Zhejiang Province, Ningbo University, Ningbo, 315211 Zhejiang People’s Republic of China

**Keywords:** Adhesion, *Lactobacillus acidophilus* NCFM, S-layer protein, Caco-2 cell, pET-28a-slpB, His-slpB fusion

## Abstract

The *slpB* gene of *Lactobacillus acidophilus* NCFM, which differs from the *slpA* gene and is silent under normal conditions, was successfully amplified and ligated to the corresponding available sites on a recombinant pET-28a vector. Then the pET-28a-slpB vector was transformed into *Escherichia coli* DH (DE3) and the fusion His-slpB protein was expressed by induction with 1 mM IPTG for 14 h at 37 °C. The resulting His-slpB protein (S_B_) had a relative molecular weight of 48 kDa. It was purified using a Ni-NTA column and was confirmed by sodium dodecyl sulfate-polyacrylamide gel electrophoresis and western blot contrastive analysis. The *slpA* protein (S_A_) from *L. acidophilus* NCFM was extracted and purified. It had a relative molecular weight of 46 kDa. Circular dichroism measurements suggested that the two S-layer proteins had a high β-sheet content and a low α-helix structure content. In an adhesion experiment, S_A_ displayed higher adhesive capability towards Caco-2 cells than did S_B_. The results suggest that these two S-layer proteins could have biotechnological applications.

## Introduction

Probiotic microorganisms, such as *Lactobacillus,* which is a normal flora found in the intestines of humans and animals (Walter et al. 2003), balance endogenous bacteria, protect the intestinal tract from competition, manufacture antimicrobial molecules, and stimulate mucosal immunity (Singh et al. 2016). The ability of *Lactobacillus* to adhere to intestinal epithelial cells is extremely important. The adhesive ability of probiotics is usually related to the immune effect of combined probiotics and their ability to interfere with the adhesion of pathogens (Ohashi and Ushida 2009). The S-layer protein of *Lactobacillus* has the ability to mediate *lactobacillus* adherence to host cells, gastrointestinal tissue and the extracellular matrix (Hynönen et al. 2002). S-layer proteins usually have a layer of lattice-like single-molecule proteins or a glycoprotein subunit of the cytoplasm outer layer varying in mass from 40 to 200 kDa (Lavermicocca et al. 2005) with regular and porous arrays displaying different types, such as oblique (p1, p2), square (p4) or hexagonal (p3, p6), of symmetry. The thickness of the S-layer protein is generally between 5 and 15 nm, and S-layer protein is smoother than the bacterial internal surface structure (Sára and Sleytr 2000). S-layer protein covers up to 70% of the total area of a microorganism (Toca-Herrera et al. 2005). S-layer proteins are found in most bacteria, such as *L. helveticus*, *L. acidophilus*, *L. bulgaricus* and *L. brevis* (Johnson et al. 2013). Most S-layer proteins can be broken down via their hydrogen bonds using high concentrations of solvents to achieve separation (Baumeister et al. 1989). The isoelectric point (pI) of most bacterial S-layer proteins is acidic, and only the pI of *Lactobacillus* S-layer proteins is alkaline (9.35–10.4), because S-layer proteins possess a high number of specific amino acids with a positive charge (Sleytr and Sára 1997). The mass fraction of S-layer proteins with strong gene expression and secretion capacity is 10–15% of the total bacterial protein (Lavermicocca et al. 2005).

Gene sequencing of S-layer protein has been a focus of research in recent years and other *Lactobacilli* have been found to carry S-layer proteins. S-layer proteins are used in the efficient expression and localization of heterologous proteins or polypeptides because of the high transcriptional efficiency of gene promoters (Lindholm et al. 2004). Owing to the high secretion efficiency of S-layer protein signal peptides, they are widely used as an efficient expression vector of heterologous strains (Novotny et al. 2005). *L. acidophilus* has two S-layer protein genes (Boot et al. 1993): *slpA* is expressive under normal conditions, while *slpB* is silent. These two S-layer proteins encoding genes are located on a chromosomal fragment of 6 kb (the slp segment) (Boot et al. 1996). *L*. *acidophilus* bacteria grown under laboratory conditions appear to have a selective advantage when they express the *slpA* instead of the *slpB* gene, because 99.7% of these bacteria had the *slpA* gene present at the slp expression site (Boot et al. 1996). It was found that *slpB* lacked promoter substructure, while there were two promoters at the upstream of *slpA* (Lightfoot et al. 2015). Therefore, it should be possible to create an expression system to express the *slpB* gene. This endeavor is important in order to analyze the biological function of the silent protein.

Increased understanding of the structure, chemistry, assembly, and genetics of S-layer proteins will enhance our ability to use them effectively in the future. The main purpose of this study was to analyze some of the features of S-layer proteins. Our specific tasks were to construct a recombinant expression vector pET-28a-slpB, and to express and purify the fusion His-slpB protein. *SlpA* protein from *L. acidophilus* NCFM was extracted and purified. Then the secondary structure and adhesion ability onto Caco-2 cells of the two S-layer proteins were compared.

## Materials and methods

### Bacterial strains

*Escherichia coli* DH10B and *E. coli* BL21 were purchased from the China Industrial Microbial Culture Collection (Beijing, China). *L. acidophilus* NCFM was purchased from the American Type Culture Collection (ATCC, Manassas, VA, USA, ATCC 700396). pET28a and pMD19T simple were obtained from Dalian Bao Biological Engineering Co. (Shandong, China).

### Construction of a recombinant pET-28a-slpB expression vector

The whole gene of *L. acidophilus* NCFM was extracted by using an Ezup Column Bacteria Genomic DNA Purification Kit (Sangon). The *slpB* gene of *L. acidophilus* NCFM was amplified taking a pair of particular primers: forward cgcCATATGGTATCTACTGTTAACGCTGCCGCTGTTAATG; reverse cgCTCGAGTTATCTAAAGTTTGCAACCTTAACGTAAGTCTTGTCAGTG. The underlined bases were *Nde*I/*Xho*I sites. The polymerase chain reaction (PCR) amplification program was 95 °C for 5 min, 95 °C for 45 s (30 cycles), 60 °C for 45 s (30 cycles), 72 °C for 60 s (30 cycles), and 72 °C for 10 min (Tan et al. 2017). The product was purified using a QuiMag Gel Micro DNA purification kit (Sangon).

The recombinant expression vector pET-28a was digested by *Nde*I/*Xho*I restriction enzymes. Then the PCR product was cloned to the expression plasmid vector pET-28a at the corresponding sites. The ligation product of vector pET-28a-slpB was transformed to *E. coli* DH10B using the calcium chloride method (Fatemeh et al. 2015). Colony filtrate was obtained and the pET-28a-slpB plasmid was extracted from the transformed cells. To verify whether the vector pET-28a had the *slpB* gene, the recombinant plasmid was digested with *Nde*I/*Xho*I restriction enzymes and then electrophoresis was run on a 1% agarose gel. The accuracy of the verification vector pET-28a-slpB was affirmed by DNA sequencing (Sangon Biotech (Shanghai)). The strain having the correct expression vector pET-28a-slpB was preserved for future use by using the glycerol stocks method (Singh et al. 2016).

### Expression and purification of the His-slpB fusion protein

To express and purify the fusion protein, the recombinant vector pET-28a-slpB were transformed into *E. coli* DH (DE3) cells. To obtain the best induction conditions and more protein, the *E. coli* DH (DE3) cells were grown in Luria–Bertani (LB) medium containing 30 μg/mL Kanamycin with shaking (220 rpm) at different temperatures (20, 37 °C), concentrations of isopropyl-β-d-thiogalactoside (IPTG) (0, 0.1, 0.25, 0.5, 1 and 5 mM) and induction times (4, 6, 8, 10, 12, 14 and 16 h). The *E. coli* DH (DE3) cells were centrifuged (6000×*g* for 10 min at 4 °C) and the cell deposit was suspended in phosphate buffer saline (PBS) solution. The sample was then sonicated for 60 circulations of 300 s (400 W) at low temperature. The suspension was centrifuged (15,000×*g* for 30 min at 4 °C) to obtain the liquid supernatant, which was put in a Ni-NTA column to purify the His-slpB. Finally, the fusion His-slpB protein was visualized using SDS-PAGE and determined by Western blot. The concentration of the purification was measured using a Coomassie Blue staining kit (Sangon).

### Western Blot assay of His-slpB

The His-slpB fusion protein was determined by SDS-PAGE. After SDS-PAGE, the gel with His-slpB fusion protein was put in the transfer box (200 mA for 1.5 h). The Poly vinylidene fluoride (PVDF) membrane was removed and placed in Tris-Buffered Saline Tween-20 (TBST) for 10 min. The PVDF membrane was then transferred to the blocking buffer and left at room temperature for 2 h. The PVDF membrane was washed with TBST 3 times (5 min/wash). The antibody was diluted 1:3000 with 3% BSA/TBS and the PVDF membrane was incubated for 12 h at 4 °C. The PVDF membrane was washed with TBST 5 times (5 min/wash). Then 3,3′,5,5′-tetramethylbenzidine (Promega) was added. The reaction was stopped by washing with distilled water when the specific protein band appeared (Zhang et al. 2016).

### Extraction and purification of *slpA* protein from *L. acidophilus* NCFM

After being sub-cultured twice, *L. acidophilus* NCFM strains were grown in 400 mL autoclaved MRS liquid medium for 18 h at 37 °C under anaerobic condition. The cell culture was centrifuged (6000×*g*, 4 °C, 10 min) to obtain the cell precipitation. Cell precipitation was washed 3 times with PBS solution. Six millilitre of 5 mol/L LiCl (pH 2.0) was added to the cell suspension, then mixed for 20 min in an ice-water bath. The liquid supernatant containing crude protein was obtained after centrifugation (10,000×*g*, 4 °C, 10 min). The crude protein was purified using Sephadex G-75 chromatography. The specimen additive included 4 mL of crude protein and 2 mL/min of elution speed by using 0.025 mol/L Tris–HCl buffer (pH9.5). Then the sample was imaged at 280 nm for ultraviolet absorption. The eluent was dialyzed against distilled water with a dialysis bag at 4 °C for 18 h. The distilled water was changed until there were no chloride ions discovered using silver nitrate. Finally, the purified protein was frozen to improve the concentration for later use.

### Secondary structure determination of two S-layer proteins by circular dichroism

Circular dichroism is often used to detect the secondary structure of a protein in solution (Kelly et al. 2005). A circular dichroism measurement was obtained using a circular dichroism analyzer (British APL company) at homeothermy. The far-UV (190–260 nm) spectra were recorded with a bandwidth of 0.5 nm and a scan rate range of 100 nm/min. The two S-layer proteins concentration of 50 μg/mL were dissolved in PBS solution. The parallel was operated three times.

### Protein modification

Three milligram of S_A_ (or S_B_) protein was soluble in 1 mL PBS solution. According to the protein molecular weight of 48 kDa, the protein molar concentration was calculated as 62.5 μM. Two milligram of FITC-NHS was dissolved in 46.6 μL DMSO and formulated as a 100 mM stock solution. One millilitre of the above protein in PBS was mixed with 31 μL of high-strength stock solution of FITC-NHS and allowed to react at room temperature for 2 h. The reaction system was added to a Millipore 3 kD ultrafiltration tube, purified by ultrafiltration 5–6 times, and centrifuged at 14,000×*g* for 10 min each. After each ultrafiltration, the liquid in the lower tube of the ultrafiltration tube was discarded and an appropriate amount of PBS solution was added to the upper tube. The protein concentration was determined according to the method recommended for the Thermo Fisher BCA Protein Assay Kit (Shanghai, China). The protein was denatured by adding 5× protein loading buffer at 99 °C for 5–10 min. Fluorescence images were taken under the FITC channel and stained with Coomassie brilliant blue to mark the position of the protein under a bright field and the two images were combined to observe the efficiency of their coupling.

### Caco-2 cell culture

Caco-2 cells were purchased from the Shanghai Institute of Cell Biology (Shanghai, China). The cells were grown in Dulbecco’s Modified Eagle’s Medium (DMEM) containing 10% fetal bovine serum (FBS) and 1% penicillin–streptomycin solution in an incubator with 10% CO_2_ at 37 °C. The culture medium was changed daily. The cells were sub-cultured every 5 days and were used for the experiment after 15 days. The concentration of the cells was regulated to 2 × 10^5^/cm^2^ and the cells were inoculated into 6-well tissue plates for adhesion assays when the cells grew to a monolayer (80–90%).

### Determination of protein concentration—Bradford method

A standard protein solution of 1 mg/mL was prepared with bovine serum albumin (BSA) as the standard protein, and a set of three repetitions were set for each group of 2–20 μg. One millilitre of Bradford working solution was added. After shaking for 2 min, absorbance was measured at 595 nm. The entire measurement process was performed within 1 h to make an A_595 nm_-BSA standard curve. The measured A_595 nm_ was substituted into the equation of the standard curve and the protein concentration of the solution could be obtained by calculation (Gabelsberger et al. 1993).

### Analysis of two S-layer proteins’ adherence to Caco-2 cells

The two S-layer proteins were modified with fluorescein isothiocyanate (FITC) and the concentrations of the purified S-layer proteins were determined using the Bradford method. Caco-2 cells were inoculated in 96-well tissue plates. After 24 h, various concentrations (1, 5, 10, 50 and 100 μg/mL) of SA (or SB) were added to each well for 2 h. The Caco-2 cells were washed 3 times using sterile PBS solution. The results of the adhesion mechanism were analyzed using a microplate reader and observed with a laser scanning confocal microscope (LSCM).

## Results

### Construction of a recombinant vector pET-28a-slpB

**Fig. 1:**
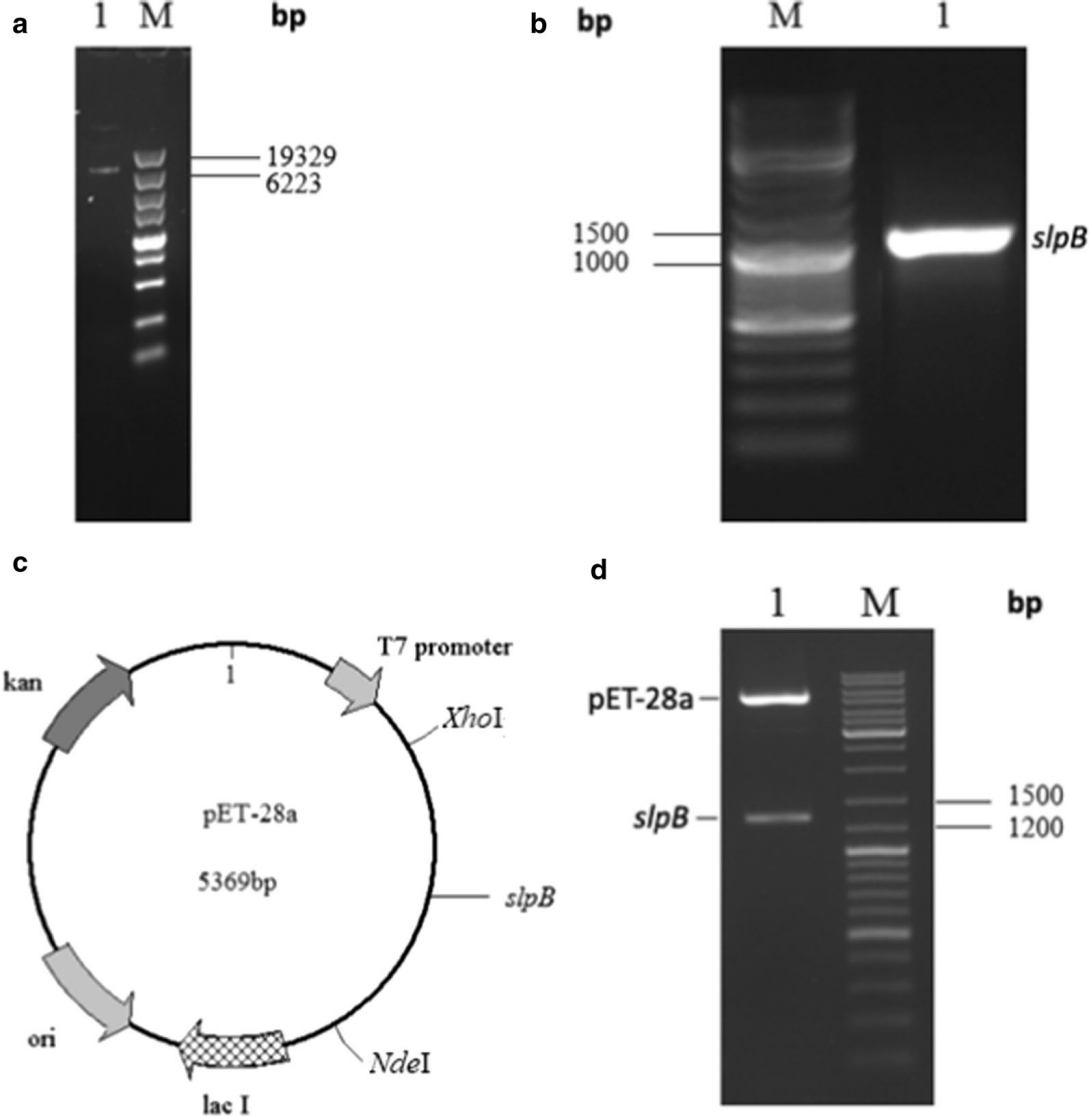
Construction of a recombinant pET-28a-slpB expression vector. **a** Total DNA of *Lactobacillus acidophilus* NCFM. **b** PCR amplification result of *slpB* gene. **c** Construction of pET-28a-slpB. **d** Identification of pET-28a-slpB by restriction enzymes

The result of agarose gel electrophoresis of the whole gene of *L.* NCFM showed that the gene was pure, complete and suitable to use to clone the *slpB* gene (Fig. 1a). The *slpB* gene was cloned using the template of the whole gene of *L.* NCFM and specific primers. Agarose gel electrophoresis showed a band at about 1300 bp, which was in accord with the NCBI gene data bank (Fig. 1b). Then the pET-28a vector was digested with *Nde*I/*Xho*I restriction enzymes and the *slpB* gene was ligated to the corresponding sites on the pET-28a vector to form a recombinant pET-28a-slpB expression vector (Fig. 1c). The recombinant expression vector pET-28a-slpB was digested by *Nde*I/*Xho*I restriction enzymes. Agarose gel electrophoresis showed bands at 5400 and 1300 bp for the vector pET-28a and *slpB* gene segments (Fig. 1d). Moreover, the results of DNA direct sequencing proved that the *slpB* gene was in the pET-28a vector; neither point mutation nor frameshift was detected. The sequencing results were analyzed using BLAST and the thrust *slpB* gene was found 100% homologous to the printed *slpB* gene nucleotide sequence (GenBank Accession Gene ID: 3252692).

### Expression and purification of recombinant His-slpB fusion protein

**Fig. 2:**
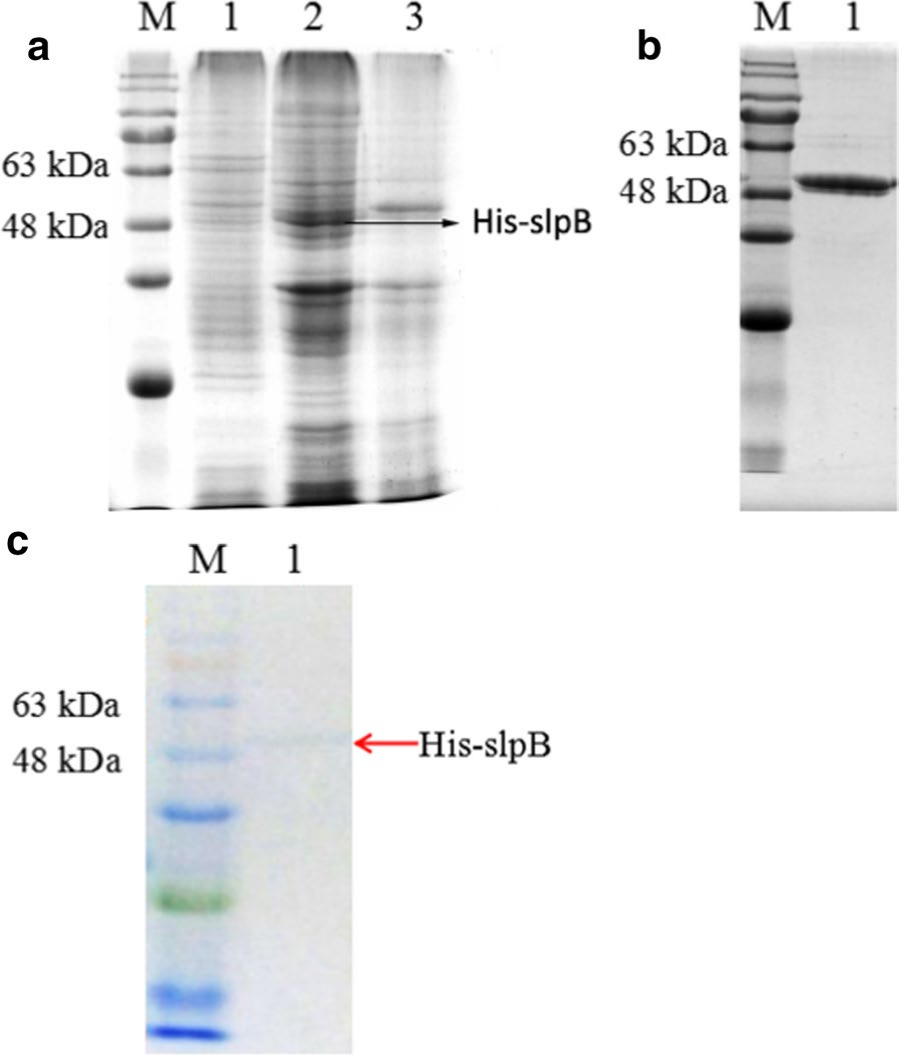
Expression and purification of the fusion His-slpB protein. **a** Expression of His-slpB fusion protein. (1) *E. coli* DH (DE3) without transformation as a negative control; (2) Induced *E. coli* DH (DE3) expression of His-slpB fusion protein; (3) Un-induced *E. coli* DH (DE3) expression of His-slpB fusion protein; **b** SDS-PAGE of purified His-slpB. **c** Western blot of purified protein

The *E. coli* DH (DE3) was used to express fusion His-slpB protein. After transformation and expression at 37 °C, 1 mM IPTG induction and 14 h induction time, the result of SDS-PAGE showed the band of the fusion His-slpB protein at about 48 kDa, which was in accord with its theoretical value (Fig. 2a, lane 2). The fusion His-slpB protein was purified with a Ni-NTA column. After purification of the His-slpB protein and SDS-PAGE analysis, a 48 kDa band of the His-slpB protein was noticed without any other proteins (Fig. 2b). To support the accuracy of the His-slpB protein analysis, Western blot using the anti-His-tag antibody demonstrated a band at about 48 kDa for the His-slpB protein (Fig. 2c).

### Extraction and purification of *slpA* protein from *L. acidophilus* NCFM

**Fig. 3:**
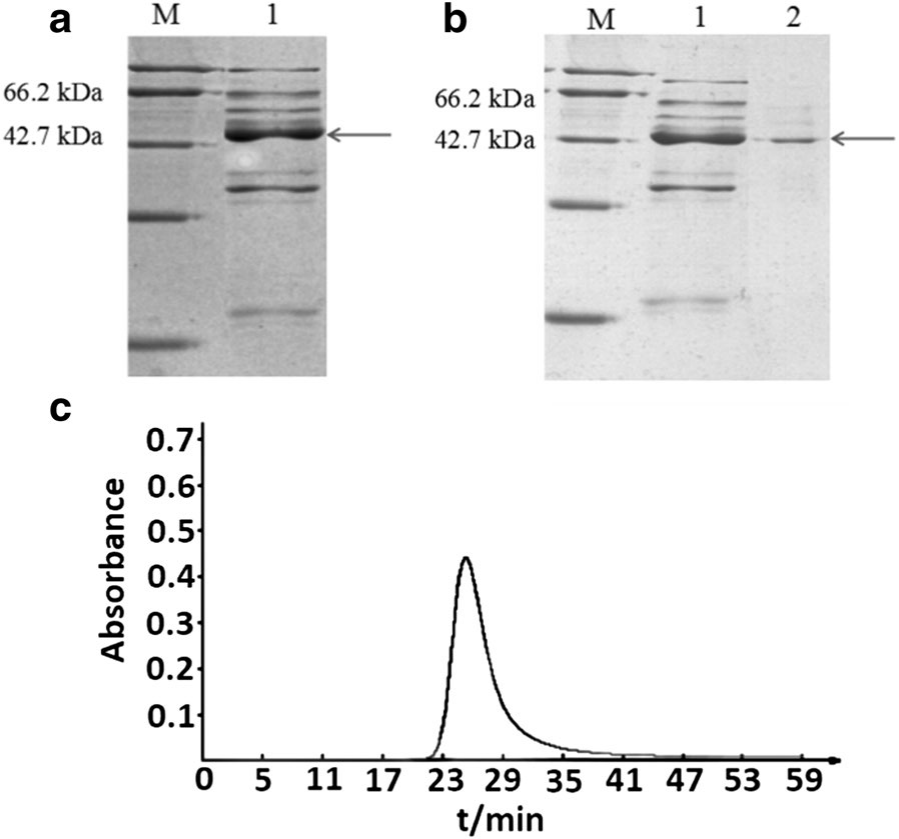
Extraction and purification of *slpA* protein from *Lactobacillus acidophilus* NCFM. **a** SDS-PAGE analysis of S_A_ extracted by acid 5 mol/L LiCl. **b** Analysis of S_A_ before and after purification. **c** Elution profile of S_A_ purified by gel chromatography

The *slpA* protein from *L. acidophilus* NCFM was named S_A_ and the His-slpB protein was named S_B_. The S_A_ was extracted from *L.* NCFM by using 5 mol/L LiCl (pH 2.0). The arrow showed a much greater protein concentration compared to the other proteins. It was crude S_A_ (Fig. 3a). Sephadex G-75 chromatography was used to purify the crude S_A_. One obvious elution peak appeared at about 26 min in the S_A_ elution profile (Fig. 3c). In the light of the electrophoretic bands of the peak, there was an obvious and singular band at 46 kDa, which meant that the S_A_ molecular mass was around 46 kDa (Fig. 3b).

### Secondary structure of two S-layer proteins using circular dichroism

**Fig. 4:**
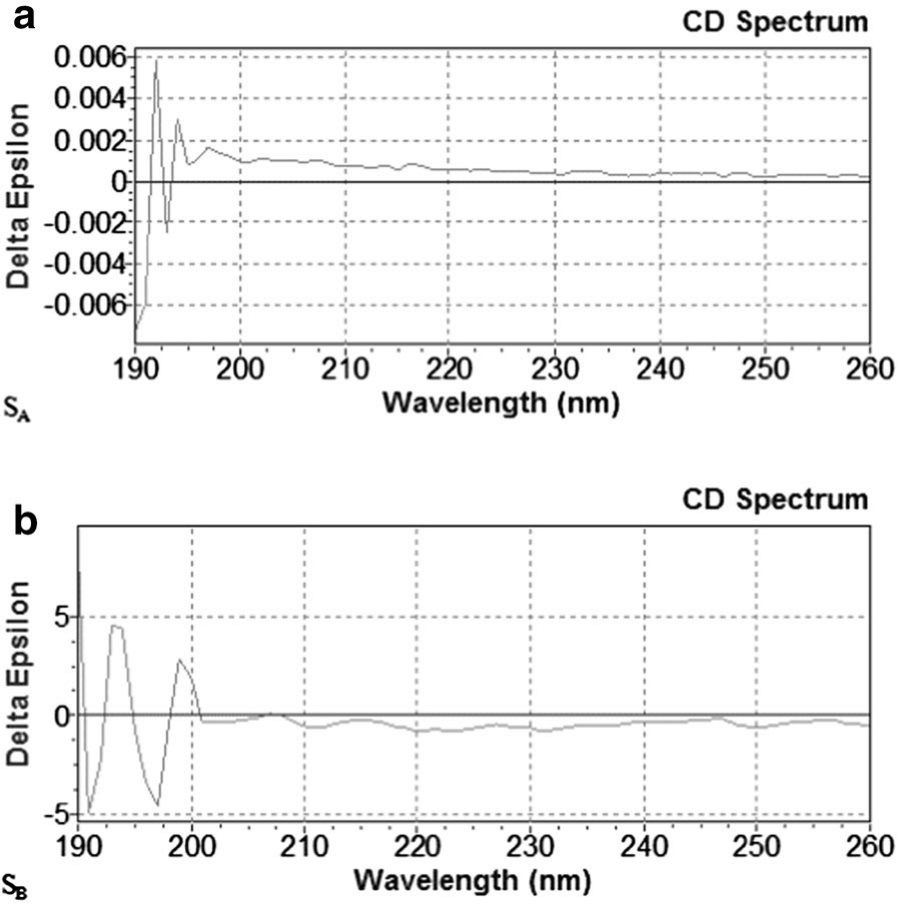
Secondary structure determination of two S-layer proteins by circular dichroism. **a** CD Spectrums of the S_A_. **b** CD Spectrums of the S_B_

CD measurements in the far UV region (190–260 nm) were made to probe the amount of secondary structure of S_A_ and S_B_ under similar experimental conditions. The amount of secondary structure of S_A_ and S_B_ is summarized in Table 1. Taken together, these results suggest that the two S-layer proteins had a relatively high β-sheet content and a low α-helix structure content (Fig. 4).

**Table 1:** The amount of secondary structure of the two S-layer proteins

S-layer protein	α-helix (%)	β-sheet (%)	β-turn (%)	Random coil (%)
S_A_	16.9	55.6	20.8	6.7
S_B_	17.9	49.3	20.2	12.6

### Comparison and analysis of the two S-layer proteins’ adherence to Caco-2 cells

**Fig. 5:**
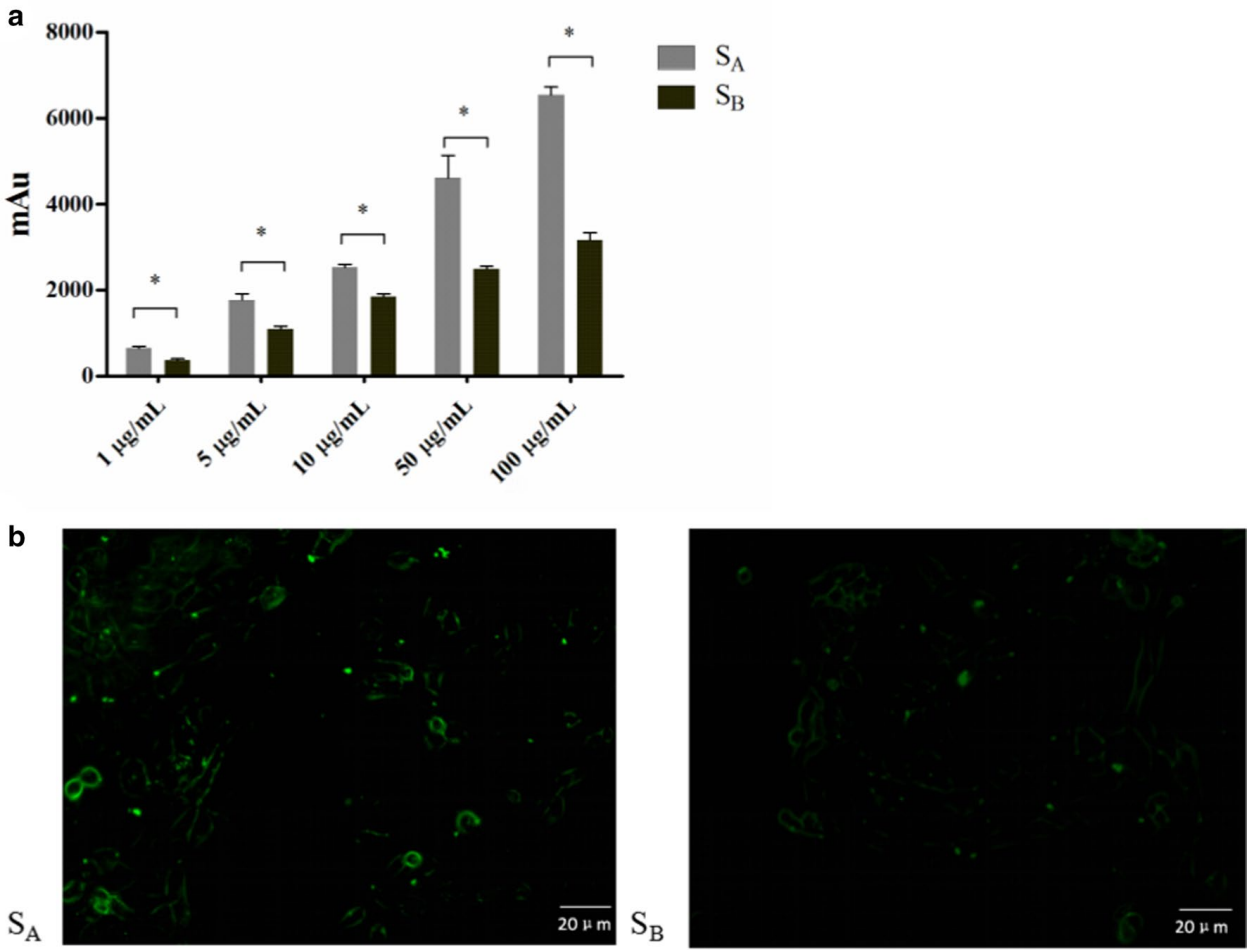
Analysis of two S-layer proteins’ adherence to Caco-2 cells. **a** Analysis of two S-layer proteins’ adherence to Caco-2 cells by microplate reader fluorescence assay. **b** Analysis of two S-layer proteins’ adherence to Caco-2 cells by laser confocal imaging

To analyze the adhesion ability of the two S-layer proteins to Caco-2 cells, a microplate reader fluorescence assay and laser confocal imaging analysis were used. S_A_ showed higher adherence ability in both the microplate reader fluorescence assay (Fig. 5a) and under laser confocal imaging (Fig. 5b) than S_B_.

## Discussion

In recent years, some *Lactobacillus* gene sequences have been elucidated. Researchers have used *E. coli* to clone and express the S-layer protein genes of *L. helveticus* and *L. brevis.* Boot et al. (1993) used phage to transfer to the *E. coli* in order to clone and express the surface protein gene of *L. acidophilus*. However, phage as a carrier has disadvantages, including unstable packaging efficiency, a complex operational method and a limited screening ability. In this experiment, we made some improvements. When the amplified product *slpB* gene was sent to DNA sequencing, the results always showed a deletion mutation. Due to the late exploration of protein expression, it is necessary to guarantee the correctness of the base sequence. Therefore, we changed the process. The Taq DNA polymerase was selected for the PCR reaction. Therefore, the *slpB* gene was connected to pMD19T(Simple) to construct a recombinant plasmid pMD19T(Simple)-slpB. T-A cloning made the template more stable and the sequencing effect was improved. In recent years, researchers have cloned and expressed the *slpA* gene, which is normally expressed in *Lactobacilli*. Studies have shown that after knocking out the *slpA* gene, the silent gene *slpB* is expressed, but the process is cumbersome (Goh et al. 2009). Although a few studies have focused on the silent gene *slpB* of *Lactobacilli*, the present study will provide important information for a better understanding of the structure and biological function of the protein.

*Escherichia coli* is usually used as a fusion protein expression system due to its simple operational method and low cost (Terpe 2006). *E. coli* BL21(DE3) is frequently used as an expression strain and contains T7 polymerase factor. T7 is a controllable expression promoter and has high specificity. When the *E. coli* BL21(DE3) reaches a certain density of bacteria, adding certain chemical substances or adjusting growth conditions can promote its expression (Sãra 2001). In this study, the expression system of the *E. coli* is self-constructed. However, there are still some imperfections in the design and more systematic research is needed to discover the rules of expression. This experiment provides a new idea in relation to directing the development of an expression system for the silent gene *slpB*.

Chromatography and electrophoresis are the most common detection methods for protein purification. In this experiment, gel filtration chromatography was used to isolate and purify the protein expressed by the *slpA* gene of *Lactobacillus*. Gel filtration chromatography can separate small and large molecules from interfering matrices (Lian et al. 2016). So, it is easy to isolate contaminants from high molecular weight interferences and widely used on the basis of the different molecular weights of proteins employed to separate the different components of a sample (Zhang et al. 2017). The method can effectively purify the target protein, ensure the biological activity of the recombinant protein, and avoid the irreversible degeneration caused by too-long treatment of the denaturant.

The proteins exist in solution mainly in the secondary structure of α-helix, β-sheet, β-turn, and random coil. In this experiment, circular dichroism spectra were used. It has been reported that the secondary structure of S-layer protein is composed of 20% α-helix, 40% β-sheet, and 5–45% random coil and β-turn (Mobili et al. 2008). The stability of the protein structure is influenced by the β-sheet; the higher the content, the higher the stability. The content of S_A_ was slightly higher than that of S_B_, indicating that it was more stable than S_B_. The glutamate and alanine contained in the protein contribute to the formation of α-helix, while valine and tyrosine facilitate the formation of β-sheet. Through the secondary structure table, it is speculated that their amino acid compositions are similar.

S-layer proteins play an important role in the regulation of adhesion to host cells, which contributes to the survival and colonization of *Lactobacillus* in the gastrointestinal tract. Some S-layer proteins can effectively prevent the adhesion of pathogens to epithelial cells. Some researchers have transferred the *slpB* gene from the site of silencing to the site of expression through gene transfer, but no cells have been detected for the expression of the *slpB* protein (Boot et al. 1996). Studies have shown that the knockout of the *slpB* gene has little effect on LTA-induced colonic inflammatory responses (Zadeh et al. 2012), and there are few reports of its adhesion. In this study, the adhesion ability of two kinds of surface proteins was studied using a Caco-2 cell model. Among the tested proteins, the protein with the higher adhesive ability was S_A_ under both microplate reader fluorescence assay and laser confocal imaging. At present, the mechanism is still not clear, and more research is needed.

## References

[CR1] Baumeister W, Wildhaber I, Phipps BM (1989). Principles of organization in eubacterial and archaebacterial surface proteins. Can J Microbiol.

[CR2] Boot HJ, Kolen CP, Noort JMV, Pouwels PH (1993). S-layer protein of *Lactobacillus acidophilus* ATCC 4356: purification, expression in *Escherichia coli*, and nucleotide sequence of the corresponding gene. J Bacteriol.

[CR3] Boot HJ, Kolen CPAM, Pouwels PH (1996). Interchange of the active and silent S-layer protein genes of *Lactobacillus acidophilus* by inversion of the chromosomal slp segment. Mol Microbiol.

[CR4] Fatemeh A, Jalil FM, Seyed Davar S, Mohammad Reza A (2015). Expression and purification of the uropathogenic *Escherichia coli* PapG protein and its surface absorption on *Lactobacillus reuteri*: implications for surface display system vaccines. Jundishapur J Microb.

[CR5] Gabelsberger J, Liebl W, Schleifer KH (1993). Purification and properties of recombinant β-glucosidase of the hyperthermophilic bacterium *Thermotoga maritima*. Appl Microbiol Biotechnol.

[CR16] Goh YJ, Azcarate-Peril MA, O'Flaherty S, Durmaz E, Valence F, Jardin J, Lortal S, Klaenhammer TR (2009). Development and application of a upp-based counterselective gene replacement system for the study of the S-layer protein SlpX of Lactobacillus acidophilus NCFM. Appl Environ Microbiol.

[CR6] Hynönen U, Westerlund-Wikström B, Palva A, Korhonen TK (2002). Identification by flagellum display of an epithelial cell- and fibronectin-binding function in the *SlpA* surface protein of *Lactobacillus brevis*. J Bacteriol.

[CR7] Johnson B, Selle K, O’Flaherty S, Yong JG, Klaenhammer T (2013). Identification of extracellular surface-layer associated proteins in *Lactobacillus acidophilus* NCFM. Microbiology.

[CR8] Kelly SM, Jess TJ, Price NC (2005). How to study proteins by circular dichroism. BBA Proteins Proteomics.

[CR9] Lavermicocca P, Valerio F, Lonigro SL, De AM, Morelli L, Callegari ML, Rizzello CG, Visconti A (2005). Study of adhesion and survival of lactobacilli and bifidobacteria on table olives with the aim of formulating a new probiotic food. Appl Environ Microb.

[CR10] Lian W, Ren F, Tang L, Dong D (2016). Analysis of polycyclic aromatic hydrocarbons in cigarette samples using gel permeation chromatography clean-up by gas chromatography–tandem mass spectrometry. Microchem J.

[CR11] Lightfoot YL, Selle K, Yang T, Goh YJ, Sahay B, Zadeh M, Owen JL, Colliou N, Li E, Johannssen T, Lepenies B, Klaenhammer TR, Mohamadzadeh M (2015). SIGNR3-dependent immune regulation by *Lactobacillus acidophilus* surface layer protein A in colitis. EMBO J.

[CR12] Lindholm A, Smeds A, Palva A (2004). Receptor binding domain of *Escherichia coli* F18 fimbrial adhesin FedF can be both efficiently secreted and surface displayed in a functional form in *Lactococcus lactis*. Appl Environ Microb.

[CR13] Mobili P, Londero A, Maria TMR, Eusébio MES, Antoni GLD, Fausto R, Gómez-Zavaglia A (2008). Characterization of S-layer proteins of *Lactobacillus* by FTIR spectroscopy and differential scanning calorimetry. Vib Spectrosc.

[CR14] Novotny R, Scheberl A, Giry-Laterriere M, Messner P, Schäffer C (2005). Gene cloning, functional expression and secretion of the S-layer protein SgsE from *Geobacillus stearothermophilus* NRS 2004/3a in *Lactococcus lactis*. FEMS Microbiol Lett.

[CR15] Ohashi Y, Ushida K (2009). Health-beneficial effects of probiotics: its mode of action. Anim Sci J.

[CR17] Sãra M (2001). Conserved anchoring mechanisms between crystalline cell surface S-layer proteins and secondary cell wall polymers in Gram-positive bacteria?. Trends Microbiol.

[CR18] Sára TP, Sleytr RK (2000). S-layer proteins. J Bacteriol.

[CR19] Singh TP, Malik RK, Kaur G (2016). Cell surface proteins play an important role in probiotic activities of *Lactobacillus reuteri*. Nutrire.

[CR20] Sleytr UB, Sára M (1997). Bacterial and archaeal S-layer proteins: structure-function relationships and their biotechnological applications. Trends Microbiol.

[CR21] Tan TS, Syed HS, Yap WB (2017). Expression of surface-bound non-structural 1 (NS1) protein of influenza virus A H5N1 on *Lactobacillus casei* strain C1. Lett Appl Microbiol.

[CR22] Terpe K (2006). Overview of bacterial expression systems for heterologous protein production: from molecular and biochemical fundamentals to commercial systems. Appl Microbiol Biotechnol.

[CR23] Toca-Herrera JL, Krastev R, Bosio V, Küpcü S, Pum D, Fery A, Sára M, Sleytr UB (2005). Recrystallization of bacterial S-layers on flat polyelectrolyte surfaces and hollow polyelectrolyte capsules. Small.

[CR24] Walter J, Heng NC, Hammes WP, Loach DM, Tannock GW, Hertel C (2003). Identification of *Lactobacillus reuteri* genes specifically induced in the mouse gastrointestinal tract. Appl Environ Microbiol.

[CR25] Zadeh M, Khan MW, Goh YJ, Selle K (2012). Induction of intestinal pro-inflammatory immune responses by lipoteichoic acid. J Inflamm.

[CR26] Zhang Y, Xiang X, Lu Q, Zhang L, Ma F, Wang L (2016). Adhesions of extracellular surface-layer associated proteins in *Lactobacillus* M5-L and Q8-L. J Dairy Sci.

[CR27] Zhang D, Wu M, Guo Y, Xun M, Wang W, Wu Z, Pan D (2017). Purification of *Lactobacillus acidophilus* surface-layer protein and its immunomodulatory effects on RAW264.7 cells. J Sci Food Agric.

